# Food Preservation: Challenges and Efforts for the Future

**DOI:** 10.3390/foods9040391

**Published:** 2020-03-28

**Authors:** Yiannis Kourkoutas, Charalampos Proestos

**Affiliations:** 1 Laboratory of Applied Microbiology & Biotechnology, Department of Molecular Biology & Genetics, Democritus University of Thrace, 68100 Alexandroupolis, Greece; 2 Laboratory of Food Chemistry, Department of Chemistry, National and Kapodistrian University of Athens, 15771 Athens, Greece; harpro@chem.uoa.gr

Microbial hazards and food oxidation have acquired substantial economical, ethical and legal importance in the food industry. Administration of a variety of food additives along with strict preservation processes are applied to suppress the development of pathogenic microorganisms and oxidation reactions, as well as to prolong the shelf life of foods. Today, there is mounting pressure on food manufacturers to either completely avoid the use of chemical preservatives or to adopt “natural” alternatives. The use of natural bioactive compounds, functional microbial starter cultures and antioxidants for “synthetic preservative-free” products are included among the latest and most successful accomplishments in the food industry.

These biopreservatives may contribute to microbial safety and antioxidant activity and may also offer organoleptic, technological, nutritional and health benefits. Such agents may provide additional advantages compared to preservatives and starter cultures currently used in food manufacturing and are expected to result in the improvement and optimization of food production processes, leading to safer and healthier products. Examples include natural bioactive compounds with antimicrobial, antioxidant and health-promoting properties, as well as microorganisms that are able to synthesize antimicrobial molecules with a positive health input.

This Special Issue aimed to present the most recent achievements of specific antimicrobial activity and/or antioxidant capacity of natural products and functional microbial cultures and to summarize the current inventions, developments and ideas in the field, with a special emphasis on the technical applications obtained within the last five years, and future perspectives.

In this context, Mei et al. [1] reviewed potential replacement of chemical antimicrobials by natural preservatives from microorganisms, plants and animals. They proposed bacteriocins and organic acids from bacteria with promising antimicrobial activities against spoilage bacteria and plant-derived antimicrobials for achieving an extension of fish shelf life and a decrease in lipid oxidation. In addition, certain algae and mushroom species may also serve as a potential source of novel natural preservatives. On the other hand, although animal-derived antimicrobials have shown considerable antimicrobial activities, their allergen risk should be considered. Natural preservatives in combination to multiple hurdles, such as non-thermal sterilization processing, modified atmosphere packaging, as well as edible films and coatings, may act beneficially in fish storage.

As the industrial domain evolves rapidly and food manufacturers are facing continuous pressure to improve both products and processes, a considerable competitive advantage can be gained by the introduction of predictive modeling in the food industry. Microbial behavior is closely related to the properties of food itself like water activity, pH, storage conditions, temperature and relative humidity. The effect of these factors on microbial growth in food systems can be predicted by mathematical models issued by quantitative studies on microbial populations. Hence, application of such algorithms may allow the prediction of shifts in cell numbers in the food chain, from the harvesting to the production stages. Microbial responses evaluated through developed mathematical models should be further validated in real cases for quantitative risk assessment [2].

Edible coatings supplemented with essential oil components are another alternative to control spoilage microorganisms. Vieira et al. [3] investigated the survival of *Listeria monocytogenes* and *Salmonella enterica* serovar Typhimurium on apples treated with edible coatings based on sodium alginate (2%) (ECs) and supplemented with essential oil components, namely eugenol (Eug) at 0.2% or in combination with 0.1% (v/v) Eug and 0.15% citral (Cit). Both pathogens were able to survive on the surface of “Bravo de Esmolfe” apple. The use of ECs in fresh-cut fruits impaired the survival of both bacterial populations over 72 h at 4 °C, while the exposure of the pathogens on apples with ECs supplemented with Eug and Cit challenged with gastrointestinal fluids significantly reduced their survival.

Finally, the effect of sugar content (0%, 0.30% and 0.60%) on the quality attributes and shelf life of dry-fermented sausages stored for 66 days containing free or immobilized *Lactobacillus casei* ATCC 393 on wheat was studied by Sidira and co-workers [4]. A drastic decrease was recorded in the numbers of enterobacteria, staphylococci and pseudomonads during ripening in all cases. Noticeably, sugar addition and the probiotic culture resulted in a significant increase of shelf life, whereas levels of *L. casei* ATCC 393 after 66 days of ripening persisted above 6 log cfu/g. Sugar addition had a positive effect on the sensory attributes; although all products were of high quality, the immobilized cells provided a distinctive characteristic aroma and a fine taste.

To conclude, the papers included in the present Special Issue summarized applications of natural bioactive compounds and beneficial microorganisms as potential alternatives in food preservation. However, as suggested by several authors, more studies are required to validate the potential of biopreservatives in real food systems.
